# Rapid Cyanobacteria Species Identification with High Sensitivity Using Native Mass Spectrometry

**DOI:** 10.1021/acs.analchem.1c03412

**Published:** 2021-10-18

**Authors:** Jaspreet K. Sound, Anna Peters, Jeddidiah Bellamy-Carter, Cecilia Rad-Menéndez, Karen MacKechnie, David H. Green, Aneika C. Leney

**Affiliations:** † School of Biosciences, University of Birmingham, Edgbaston, Birmingham B15 2TT, U.K.; ‡ Scottish Association for Marine Science, Argyll PA37 1QA, U.K.; § Culture Collection of Algae and Protozoa (CCAP), Scottish Marine Institute, Oban PA37 1QA, U.K.

## Abstract

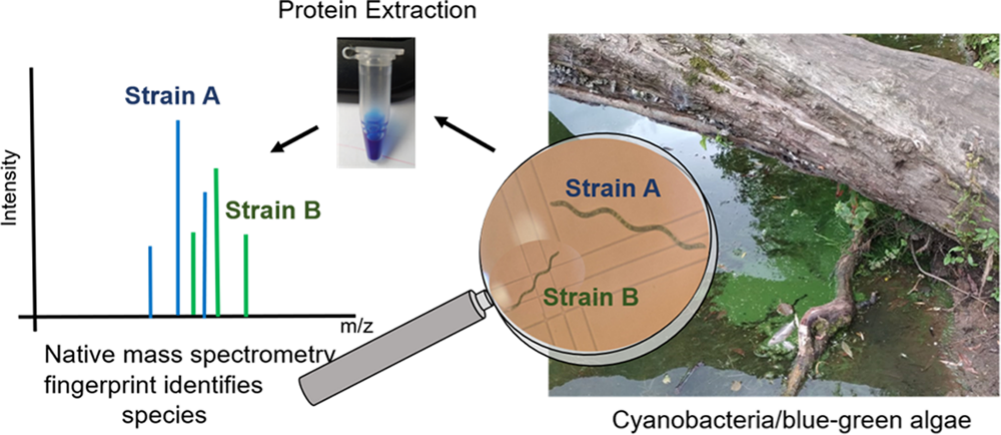

Cyanobacteria have evolved over billions of years to adapt and survive in diverse climates. Environmentally, this presents a huge challenge because cyanobacteria can now rapidly form algae blooms that are detrimental to aquatic life. In addition, many cyanobacteria produce toxins, making them hazardous to animals and humans that they encounter. Rapid identification of cyanobacteria is essential to monitor and prevent toxic algae blooms. Here, we show for the first time how native mass spectrometry can quickly and precisely identify cyanobacteria from diverse aquatic environments. By monitoring phycobiliproteins, abundant protein complexes within cyanobacteria, simple, easy-to-understand mass spectral “fingerprints” were created that were unique to each species. Moreover, our method is 10-fold more sensitive than the current MALDI-TOF mass spectrometric methods, meaning that cyanobacteria can be monitored using this technology prior to bloom formation. Together, the data show great promise for the simultaneous detection and identification of co-existing cyanobacteria *in situ*.

Blue-green algae, also known as cyanobacteria, present huge environmental problems due to their rapid formation of algae blooms.^1^ Algae blooms can deplete the oxygen supply and reduce light levels in lakes and oceans, killing fish and other aquatic organisms.^2^ In addition, cyanobacteria can produce hazardous toxins, such as hepatotoxins, that make these blooms harmful to animals and humans.^3^ Moreover, cyanobacteria have evolved for billions of years to survive and thrive in diverse climates, including extreme thermal, pH, and saline environments,^4^ showing that these harmful algae blooms (HABs) can present environmental problems worldwide. Cyanobacterial toxins vary significantly in nature and abundance between species, making methods for rapid and sensitive identification of these species essential. Indeed, the early detection of toxic species is critical to ensure these HABs are prevented prior to them contaminating water sources.

Traditionally, cyanobacterial identification relied upon light microscopy to differentiate species and classify them according to their morphology.^5^ Due to their high biodiversity, however, many cyanobacteria have very similar morphology making them indistinguishable using microscopy techniques, especially in the case whereby multiple species with similar morphologies co-exist. Nowadays, additional molecular analysis of the 16S rRNA genes and internal transcribed spacer regions within the DNA is required for taxonomic identification.^6^ This is straightforward if an axenic culture can be obtained for the species of interest. Yet, cyanobacteria associate with other microorganisms. Thus, obtaining axenic cyanobacterial cultures is a difficult and time-consuming process and can be almost impossible in cases whereby the symbiotic relationship between the cyanobacteria and microorganism is strong. Metagenomics has been tremendously successful at overcoming this hurdle, enabling individual genomes to be sequenced from microbial consortia.^7^ Yet, analyzing metagenomics data requires expertise and as such is not widely done. Moreover, in some cases, 16S rRNA alone is not capable of distinguishing between morphologically distinct species such as *Microcystis* species.^8,9^ Indeed, in some cases, cyanobacteria can exchange nonessential genes (genes that do not encode essential protein complexes) that are beneficial for local adaptation.^10^

An alternative to genome sequencing is to instead identify cyanobacteria from their unique functional protein components. Matrix-assisted laser desorption/ionization time-of-flight mass spectrometry (MALDI-TOF MS) has taken strides in this area, being routinely used to identify various bacteria,^11^ fungi,^12^ and protozoa^13^ even in clinical settings. It is rapid and easy-to-use, yet has seldom been applied to identify cyanobacteria. Nevertheless, in 2015 MALDI-TOF MS was first applied to characterize mixtures of microalgae,^14^ and in 2016, Lu and coworkers were able to differentiate cyanobacterial strains using MALDI-TOF MS by extracting their ribosomal proteins and comparing the “fingerprints” of these ribosomal proteins within species.^15^ Intact cell MALDI-TOF MS has also been used to classify *Chroococcidiopsis* cyanobacteria, with ∼35 proteins identified,^16^ and distinguish *Microcystis aeruginosa* NIES-298 from *Synechocystis* sp. PCC 6803.^17^ However, due to the number of proteins and spectral features identified using intact cell MALDI-TOF MS, interpreting the data can be complicated and require expertise to determine whether the spectral differences correlate to the different species present or varied protein abundances within the species of algae present. In addition, most proteins within cyanobacteria have masses in the range of 2–20 kDa making spectral overlap common. Moreover, many of these proteins identified will have identical protein sequences between species, thus making them indistinguishable by this MALDI-TOF MS approach. Yet, the speed, precision, and availability of mass spectrometry as a technique can still be exploited. More simplistic mass spectrometric methods are in demand.

Here, we developed a simple native mass spectrometry (MS) approach that additionally incorporates the advantages of MALDI-TOF MS technology in its speed and ease of use. Native MS relies upon the preservation of proteins and protein complexes into the gas phase, enabling them to be detected in their biologically functional state.^18^ Native MS can, therefore, be used to simplify mass spectra because when multiple proteins are combined together to form protein complexes, their masses almost always become unique, and thus the spectral overlap is minimal. Moreover, using recently developed high-resolution instruments,^19^ only small amino acid substitutions in one protein subunit out of the entire protein complex is required to distinguish different protein complexes, and thus one species from another. Our approach takes advantage of the abundance of the core photosynthetic components, termed phycobilisomes, within cyanobacteria.^20^ As much as 50% of the soluble protein mass in the cyanobacterial cell constitutes phycobiliproteins,^21^ which are themselves protein complexes within the phycobilisomes. Phycobiliproteins are readily amenable to native MS analysis, and as such, this has been elegantly demonstrated by several research groups.^22−27^ Interestingly, we noted that virtually all the phycobiliprotein subunit amino acid sequences in the cyanobacteria genome are different, meaning that their protein mass could, in theory, be readily distinguished using any electrospray-ionization MS method. Comparing the ∼30 annotated protein sequences for the phycobiliproteins, phycocyanin, and allophycocyanin, in the Swiss-Prot database,^28^ only one or two instances exist whereby the alpha or beta subunits of phycocyanin or allophycocyanin are identical in a protein sequence and would give rise to an identical protein mass spectrum (Table S1). However, when looking at the protein complex level using native MS, all species would produce unique mass spectra because both the alpha and beta sequences of both phycocyanin and allophycocyanin are either not simultaneously conserved or multiple variations of phycocyanin subunits are present that are not conserved between species. For example, *Synechococcus* sp. (PCC 6301) has a phycocyanin alpha subunit with the same mass as that of *Synechococcus elongatus* (PCC 7942). However, *Synechococcus* sp. (PCC 6301) has also been reported to contain another phycocyanin alpha subunit (C-phycocyanin-2 alpha) that is not present in *S. elongatus* (PCC 7942) (Table S1). Moreover, strains that look morphologically identical have clear mass differences. For example, the phycocyanin dimers from strains WH7803, WH8103, and WH8020 in *Synechococcus* sp. have the molecular weights 35,229, 35,278, and 35,425 Da, respectively. Here, we show that native MS can indeed uniquely identify cyanobacterial species. Through native MS analysis of cyanobacterial cell lysates, we observe simple mass spectral “fingerprints” corresponding to the unique phycobiliprotein protein complexes within cyanobacteria. Due to the high resolution afforded by the Orbitrap mass analyzer, each species detected was baseline resolved from one another with no spectral overlap detected, meaning each cyanobacterial species could be identified from within a nonaxenic culture. Finally, we compare the sensitivity of the native MS method for cyanobacterial detection. Our data shows that with relatively small volumes (∼50 mL), cyanobacteria can be identified prior to algae bloom formation at levels equivalent to fluorescence spectroscopy techniques while using 10-fold less biomass than existing MALDI-TOF MS approaches. Overall, the data show the exceptional promise that native MS could have for the rapid identification of cyanobacterial species *in situ*.

## Methods

### Chemicals and Reagents

All chemicals were purchased from Thermo Fisher Scientific unless otherwise stated. Purified allophycocyanin used in the native MS experiments was purchased from Sigma-Aldrich.

### Algae Strains

The strains of microalgae used in this study are shown in Table 1.

**Table 1:** Origin of Cyanobacterial Species Used in Study^a^

species	CCAP identification	origin
*Spirulina major*	1475/3	Brackish; Norfolk, England, UK
*Coccochloris elabens*	1413/1	Brackish; San Francisco, USA
*Spirulina subsalsa*	1475/1	Brackish; Norfolk, England, UK
*Gloeocapsopsis crepidinum*	1425/1	Marine; Algarve, Portugal
*Nodularia harveyana*	1452/1	Marine; unknown
*Chroococcus* sp.	1412/6	Marine; Colonsay, Scotland, UK
*Oscillatoria nigroviridis*	1459/9	Marine; Suffolk, England, UK
*Arthrospira maxima*	1475/9	Hypersaline; Lake Chad, Africa
*Euhalothece* sp.	1421/1	Hypersaline; Qabar-Onn Lake, South Libya

a The most common species names are given, for synonyms, see Table S2.

### Algae Growth

The strains were provided by the Culture Collection of Algae and Protozoa (CCAP) in Oban, Scotland. All strains were grown on 50:50 artificial seawater: The blue-green medium (ASW/BG)^5,29−31^ on liquid was kept at 20 °C under a 12:12 h light/dark regime [25–30 μmol(photons)/m^2^ s]. The cultures were grown for 2 weeks before analysis.

### Algae Lysis and Phycocyanin Extraction

For algae lysis, 50 μL of each of the fresh cyanobacterial cultures were taken separately. The cyanobacteria aliquots were then centrifuged at 16,000 rcf (*g*) for 2 min and any residual media were removed. The algae pellet was lysed by the addition of an equal volume of milliQ water and subjected to three freeze–thaw cycles. In most cases, a blue color became immediately visible corresponding to algae cell lysis and the release of phycocyanin/allophycocyanin. If no blue color was observed, sonication was additionally performed in bursts of 1 min intervals until the blue color was observed. After a further round of centrifugation at 16,000 rcf (*g*) for 2 min, the supernatant was removed from the cell debris and buffer exchanged into 100 mM ammonium acetate (pH 6.8) using an Amicon Ultra 0.5 mL centrifugal concentrator with a 30 kDa MWCO (Merck Millipore). The algae lysates containing phycocyanin and allophycocyanin were analyzed immediately by native MS, but if required could also be stored for short periods at −20 °C in the dark.

### Species Identification Experiments

The total protein concentrations of the extracts from *Spirulina major*, *Coccochloris elabens*, *Gloeocapsopsis crepidinum*, *Nodularia harveyana*, *Spirulina subsalsa*, and *Oscillatoria nigroviridis* were determined using a DS-11 spectrophotometer (DeNovix) measuring at 280 nm using an extinction coefficient of 1 (mg/ml)^−1^ cm^–1^. The extracts were mixed at an equal ratio (approximately 0.065 mg/mL final concentration) and immediately analyzed by native MS.

### Allophycocyanin Detection Limit Experiments

Purified allophycocyanin was buffer exchanged into 100 mM ammonium acetate (pH 6.8) using an Amicon Ultra 0.5 mL centrifugal concentrator with a 30 kDa MWCO (Merck Millipore). The concentration of allophycocyanin was determined from its absorbance at 652 nm, measured using a DS 11 spectrophotometer (DeNovix), and assuming an extinction coefficient of 700,000 M^–1^ cm^–1^.^32^ From a stock solution of 0.72 g/L, allophycocyanin was diluted into 10 mM ammonium acetate to a concentration of 50, 10, 5, 2.5, and 1.26 mg/L and the samples were analyzed by native MS. The 50 and 5 mg/L allophycocyanin solutions were additionally monitored by UV–vis spectroscopy over a range of 220–750 nm, taking readings at 1 nm regular intervals.

### Detection Limit of Species Identification

*Arthrospira maxima* (CCAP 1475/9) was grown as stated above. The cells were visualized using a YS100 microscope (Nikon) (Figure S3), and cell counting was performed using a hemocytometer (Bürker-Chip, NanoEnTek). The number of cells per filament was manually counted for 15 individual filaments to produce an average of 40 ± 5 cells/filament which was then used to determine the overall cell count from the number of filaments counted in each sample. To determine the detection limit of species identification by native MS, *A. maxima* at a cell count of 500,000 ± 140,000 cells/mL was serially diluted with ASW/BG media in 10-fold steps to a final concentration of 5000 cells/mL. Volumes between 1 and 50 mL of the *A. maxima* dilutions were collected and centrifuged at 16,000 rcf (*g*) for 5 min and residual media were removed. 50 μL of milliQ water was added to each algae pellet. As described above, the algae pellets were lysed and the resulting supernatant was buffer exchanged into 100 mM ammonium acetate (pH 6.8). The allophycocyanin and phycocyanin concentration of the lysates was determined by measuring the absorbance at 620 and 650 nm, respectively, using a DS-11 spectrophotometer (DeNovix), and the equations defined in Bennett and Bogorad (1973).^33^ Lysates were stored at −20 °C prior to native MS analysis.

### Native Mass Spectrometry

MS experiments of the cyanobacterial lysates were performed on a Q-Exactive HF instrument (Thermo Fisher Scientific) coupled to a Triversa NanoMate (Advion) nano-electrospray source. Customized instrument control software, which gave access to trapping gas, mass resolution, *m*/*z* range, and quadrupole isolation parameters, was supplied by Thermo Fisher Scientific. A positive ionization mode was used with a voltage between 1.7 and 1.8 kV, and a gas pressure of 0.3 psi was applied. For MS experiments involving purified allophycocyanin, nano-eletrospray was performed using borosilicate glass capillaries pulled in-house using a P-1000 pipette puller (Sutter Instrument), and gold-coated using a sputter coater (Agar Scientific Ltd.). For all experiments, the source temperature was set at 250 °C, in-source dissociation at 0 V, S-lens RF at 100, and trapping gas pressure set to 5. All mass spectra were acquired with a resolution of 7500. Typically, an extended mass range of up to 8000 *m/z* was used with an isolation window set to 1000 *m/z* when required. The automatic gain control target and maximum ion injection time were optimized for each experiment between 1–5 × 10^6^ and 50–100 ms, respectively. Where the intensity is noted, 1 min of acquisition was combined to produce the resulting mass spectrum.

For MS experiments to determine the detection limit for the *A. maxima* species, an Orbitrap Eclipse Tribrid mass spectrometer (Thermo Fisher Scientific) coupled to nano-electrospray was used in the positive ionization mode at voltages between 1 and 1.5 kV. NanoESI was performed using borosilicate glass capillaries as detailed above. The instrument was calibrated using a positive-ion mode FlexMix (Pierce, Thermo Fisher Scientific). The source temperature was set at 250 °C, in-source dissociation at 0 V, and S-lens RF at 120%. All mass spectra were acquired in an intact protein mode at a high pressure with a resolution of 7500 and a mass range of 1000–8000 *m/z*. The automatic gain control target was set to 100% and maximum ion injection time was set to 100 ms.

### Data Processing

All mass spectra were processed using X-Calibur 4.1 (Thermo Fisher Scientific) and deconvoluted where appropriate using UniDec software *v*4.2.2.^34^ Predicted masses of the allophycocyanin and phycocyanin dimers and hexamers for each species were calculated from the amino acid sequences of the allophycocyanin alpha, alpha-B, and beta chains and phycocyanin alpha and beta chains were determined by metagenomics. Associated post-translational modifications were identified using the UniProt database^28^ and the calculated masses were adjusted to include the addition of phycocyanobilin (+585.7 Da), the removal of the initiator methionine (−131.2 Da), and the modification of Asp72 to N4-methylAsp72 (+14.0 Da) where appropriate (see Supporting Information for more details). The measured mass error on the experimentally determined molecular weight, and the percentage error between the theoretical and experimentally determined molecular weights were used to verify the presence of the allophycocyanin and phycocyanin complexes (Table S2), whereby percentage errors of <0.1% indicated the presence of the corresponding protein complex.

## Results and Discussion

### Cyanobacteria Show Simple Protein Spectral Fingerprints

**Figure 1:**
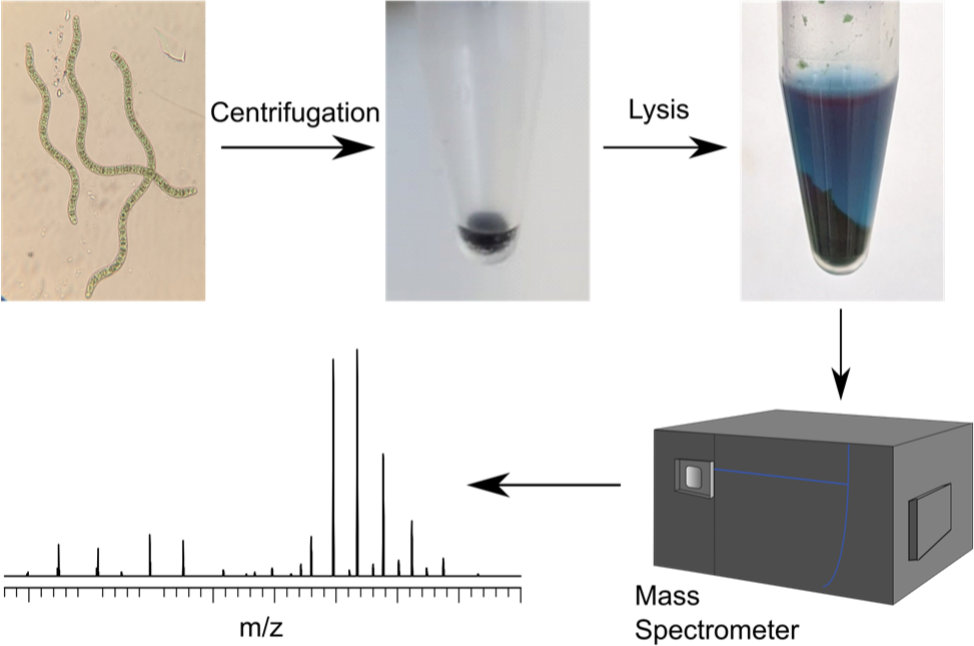
Workflow of native MS analysis of cyanobacterial lysates from fresh cyanobacterial cultures. First, the cyanobacteria biomass is pelleted, then pure water is added followed by freeze–thaw cycles and sonication to lyse the cells. Next, the lysate is buffer exchanged using a 30 kDa molecular weight filter and subsequently analyzed by native MS.

**Figure 2:**
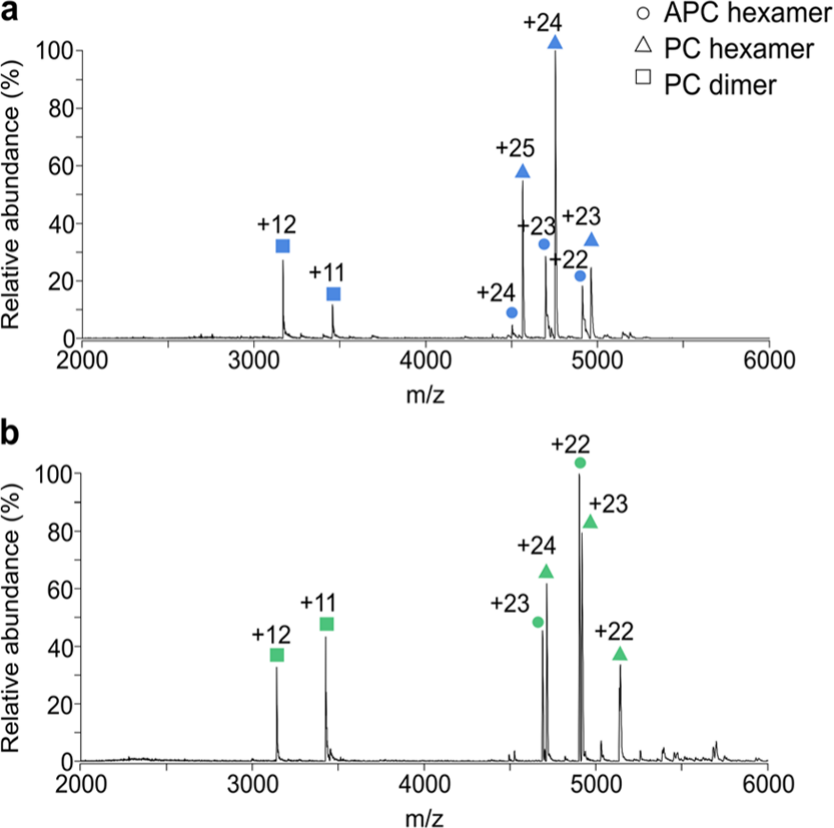
Native mass spectra show simple species dependent ”fingerprints”. Native MS of lysates from *C. elabens* (CCAP 1413/1) (a), and *O. nigroviridis* (CCAP 1459/9) (b), showing the allophycocyanin (APC) hexamer (circle), the phycocyanin (PC) hexamer (triangle), and the phycocyanin (PC) dimer (square) as the predominant protein complexes detected.

For rapid identification of cyanobacteria, a simple sample preparation approach was taken whereby cyanobacteria were lysed in ultrapure water, low molecular weight proteins were removed by passing the lysate through a molecular weight cut-off membrane filter, and the species were subsequently analyzed by native MS (Figure 1). Nine species of cyanobacteria from brackish, marine, and hypersaline environments were taken for analysis (Table 1). Despite the high number of proteins and protein complexes within cyanobacteria, simple, easily annotatable mass spectra were observed (Figure S1). Two representative species, *C. elabens* (CCAP 1413/1) and *O. nigroviridis* (CCAP 1459/9), are shown in Figure 2. Interestingly, the predominant peaks in the mass spectra correspond to phycobiliprotein complexes derived from the phycobilisome; cyanobacteria’s essential photosynthetic complex. In both cases, hexameric phycocyanin and allophycocyanin were identified with a mass deviation of less than 70 Da (or less than 0.07%) compared to their theoretically predicted masses based on the protein sequence (Table S2). These phycobiliprotein complexes correspond to the building blocks that assemble to form the intact phycobilisome. Dimeric phycocyanin was also observed, showing phycocyanin is likely in dynamic equilibrium between its dimeric and hexameric components (Figure S2). By looking at these high molecular weight complexes, we show that the mass spectra are highly simplified compared with previous MALDI-TOF MS spectra, paving the way toward species identification with relative ease. Indeed, due to the differences in protein sequences of phycobiliproteins between cyanobacterial species, the masses of both the allophycocyanin and phycocyanin complexes can clearly be distinguished (Table S1). Moreover, despite the small mass difference (174 Da for the allophycocyanin hexamer when comparing the species used in Figure 2), the high resolution afforded by the native MS instrumentation means that these species, if present in a mixture, could clearly be distinguishable. Thus, the native MS data would serve as an algae species “fingerprint” for use in quick species identification.

### Phycobiliproteins are Detected at Levels Lower than Those Measured by UV–Vis Spectroscopy

**Figure 3:**
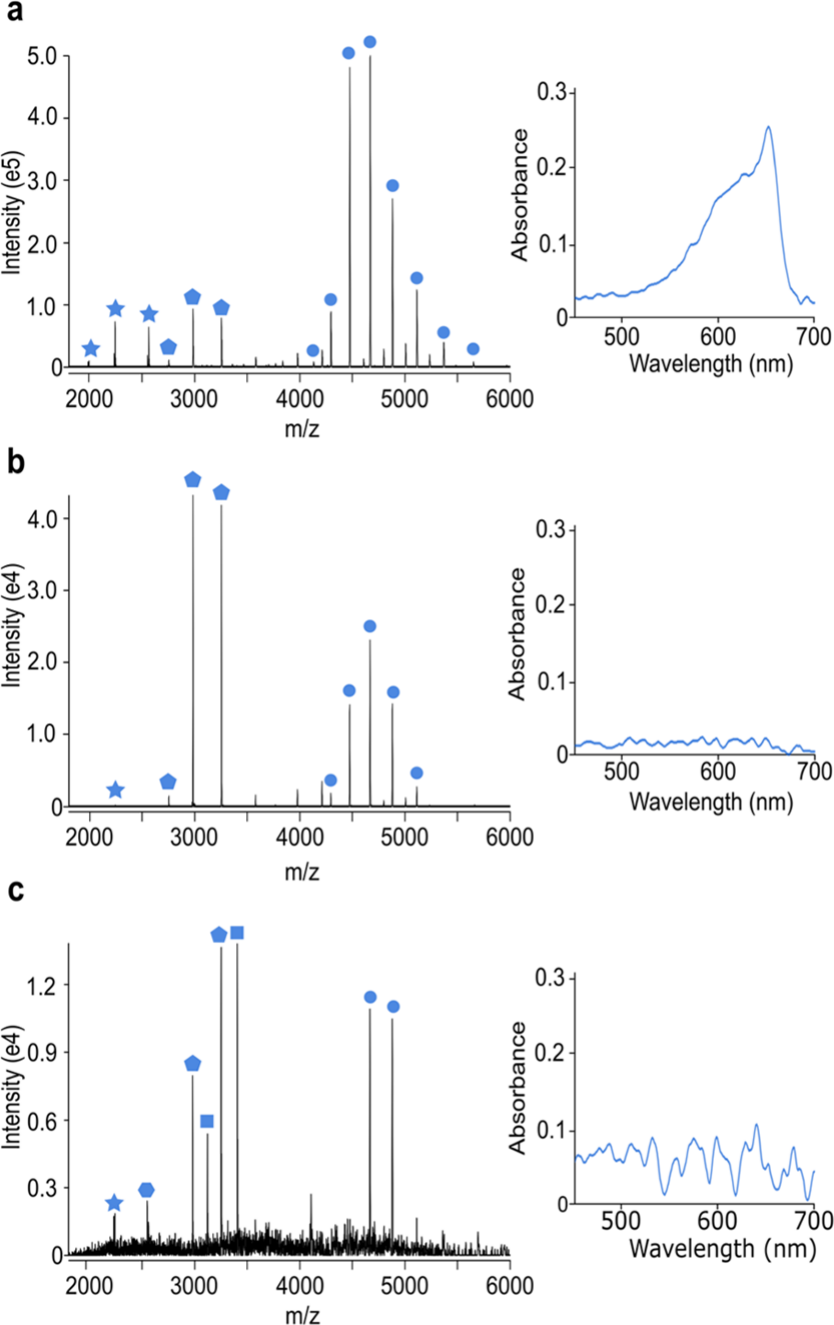
Native MS detects species at lower levels than UV–vis spectroscopy. Native mass spectra and UV spectra of 50 mg/L allophycocyanin protein standard (a), 5 mg/L allophycocyanin protein standard (b), and *Arthrospira maxima* lysate (CCAP 1475/9) (c), prepared from 50 mL of a 5000 cells/mL culture of *Arthrospira maxima* (∼250,000 cells total). The allophycocyanin hexamer (circle), allophycocyanin dimer (pentagon); phycocyanin dimer (square); allophycocyanin α monomer (hexagon) and allophycocyanin β monomer (star) are highlighted.

To control HABs, cyanobacteria would ideally be identified prior to bloom formation. This requires continuous monitoring of water sources using technology capable of detecting cyanobacteria at low levels. Absorbance measurements based on the properties of phycobiliproteins are one common way to monitor HABs. However, when phycobiliprotein levels are low, HABs are frequently undetected due to their lack of spectrophotometric signals at these concentrations. Thus, to see how our method compares to existing technology and how it could be used in an environmental setting, we next compared the sensitivity of our native MS method to UV–vis spectroscopy methods. Purified allophycocyanin, one of the predominant protein complexes within algae, was diluted stepwise to levels undetectable by UV–vis spectroscopy. In contrast to 50 mg/L (Figure 3a), at a concentration of 5 mg/L, no spectroscopic reading could be obtained at 652 nm corresponding to allophycocyanin (Figure 3b). However, peaks were readily detected in the mass spectrum corresponding to the allophycocyanin hexamer. Moreover, allophycocyanin could even be detected at 1.26 mg/L (Figure S2), that is, up to fivefold lower than the detection limit as measured on our UV–vis spectroscopy equipment. It is important to note that phycobiliprotein complexes are dynamic and thus, although consistently detected, the oligomeric status of the protein complexes may change upon lowering the concentration prior to analysis.

Fluorometers are significantly more sensitive than UV–visible spectrophotometers for measuring algae blooms, detecting phycocyanin concentrations of less than 1 μg/L.^35^ This concentration is significantly lower than our concentrations used for pure allophycocyanin (∼1000 μg/L). However, it should be noted that we are performing this analysis on less than 5 μL of sample. Thus, if we instead took 50 mL of a 1 μg/L phycocyanin concentration sample from a lake and concentrated it down to 5 μL ahead of analysis (a step already incorporated into our fast sample preparation procedure), it would also fall readily in the detection range of native MS. To verify this, we next took a fresh sample of *A. maxima* and diluted it to a cell count of approximately 5000 cells/mL; well below the HAB limit of 20,000 cells/mL as defined by the World Health Organization (WHO),^36^ and 50 mL taken for native MS analysis (Figure 3c). Allophycocyanin and phycocyanin protein complexes were clearly observed showing that native MS is sensitive enough to detect the cyanobacterial species prior to HAB formation and can be used in conjunction with fluorescence-based methods. Moreover, using native MS, only a total biomass of 2.5 × 10^5^ cells was required for detection compared to 2 × 10^6^ cells reported for a recent MALDI TOF MS method^37^ highlighting the sensitivity of native MS technology.

### Native MS Discriminates between Multiple Cyanobacterial Species

**Figure 4:**
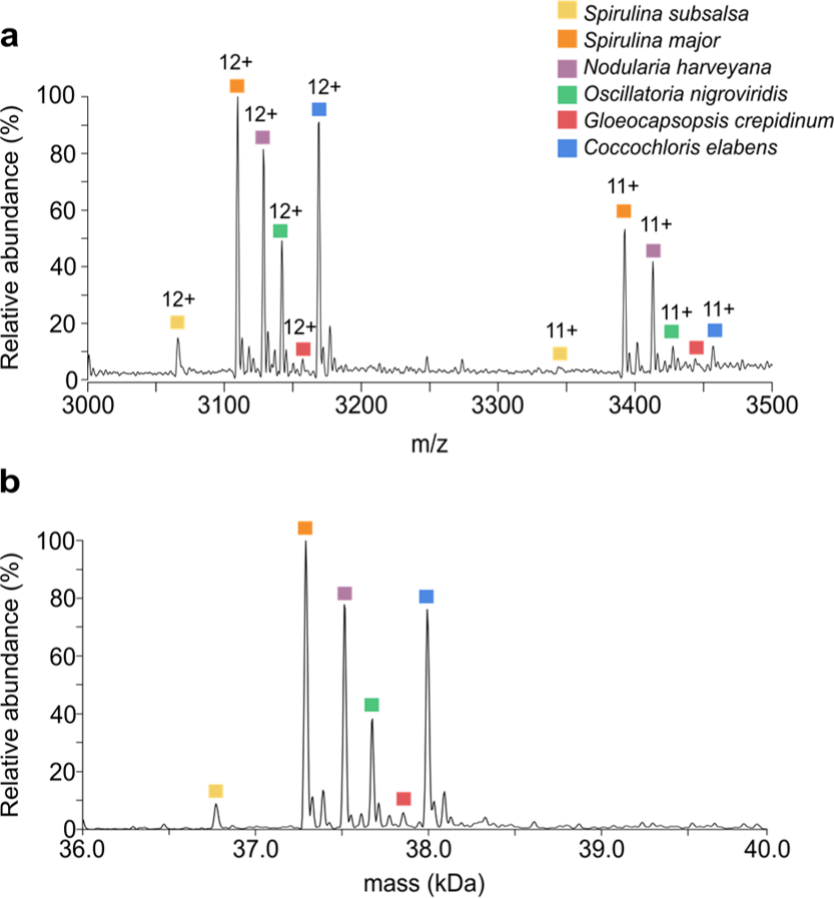
Cyanobacterial species can be detected from nonaxenic cultures. Native mass spectrum (a) and deconvoluted mass spectrum (b) of a mixture of six cyanobacterial species [*S. subsalsa* (CCAP 1475/1), *S. major* (CCAP 1475/3), *N. harveyana* (CCAP 1452/1), *O. nigroviridis* (CCAP 1459/9), *G. crepidinum* (CCAP 1425/1), and *C. elabens* (CCAP 1413/1)].

Finally, we further extended our native MS approach to the analysis of mixtures of cyanobacteria. Moreover, cyanobacteria rarely exist axenically in nature and instead co-exist with other species. Six different species of cyanobacteria were mixed and their corresponding dimer mass spectrum and deconvoluted (zero charge) spectrum are shown in Figure 4a,b, respectively. As predicted, all phycocyanin dimeric complexes had a unique mass and thus could readily be distinguished by native MS (Figure 4a). These phycobiliproteins were again the most abundant protein complexes detected throughout all the species identified, and thus even when combined, the individual mass spectral “fingerprints” are easy to extract and the presence of each species could be readily confirmed.

## Conclusions

Monitoring cyanobacterial blooms is critical to ensure that urgent action can be taken before toxic species reach dangerous levels; preventing wider environmental problems. For this, both rapid detection of species and correlation with toxin production are needed. Here, we show how native MS can be used to rapidly identify cyanobacterial species with ease. The method capitalizes on cyanobacteria’s photosynthetic ability which is enriched highly when cyanobacteria populations are rapidly expanding. We show that by analyzing only the intact protein complexes within cyanobacteria simple mass spectra can be obtained that are unique to each cyanobacterial species. Furthermore, we show that multiple species present within a mixture can be rapidly identified, highlighting the applicability of native MS to cyanobacterial identification in real water samples.

Although an exciting first step in the use of native MS to identify cyanobacterial species, more work needs to be done before this can be routinely used to analyze field samples. Due to the current lack of genome sequencing available for cyanobacteria, we would first need to create a large database of native mass spectra for all the different cyanobacteria species known. The cyanobacteria field sample could then be compared to this library and a metric defined based on the match between the spectra. A spectrum match with 0.01% mass accuracy would validate the presence of a certain species in a water sample. This data analysis involved could be automated and in doing so made accessible for nonspecialists. Indeed, it is only with the future analysis of nonaxenic cultures that we can truly validate the approach for the detection of species with vastly different abundances. Although with the advances in the dynamic range of mass spectrometers, there is no doubt that the instruments are capable of this type of quantitative native MS analysis.

Overall, upon comparison of native MS data to spectroscopic data, we show that native MS is a highly sensitive technique that can be used in parallel with current in-lake fluorometric readouts that monitor HABs. Interestingly, postbloom formation, phycocyanin can also be frequently seen on lake surfaces, thus although not investigated here, our method can also be used to identify the species from which the released phycocyanin originated.^38^ However, we believe the pinnacle of the native MS methodology described lies in species identification prior to algae bloom formation, ensuring nontoxic blooms are left untargeted while toxic blooms are kept at bay. Indeed, correlating cyanobacteria species with toxin production is the next critical step for HAB research.

## Supporting Information Available

Additional methods, cyanobacteria images, and mass spectrometry data (PDF)
