# Supplementary material for: Rapid Cyanobacteria Species Identification with High Sensitivity Using Native Mass Spectrometry
Source: Anal Chem. 2021 Oct 18;93(42):14293–9. doi: 10.1021/acs.analchem.1c03412 (PMC8552214; doi:10.1021/acs.analchem.1c03412)
Supplement: Supplementary file 1 — ac1c03412_si_001.pdf [file ac1c03412_si_001.pdf]

Supplementary Information

Rapid cyanobacteria species identification with high sensitivity using native mass spectrometry

Jaspreet K. Sound[1], Anna Peters[1], Jeddidiah Bellamy-Carter[1], Cecilia Rad-Menendez[2,3], Karen MacKechnie[2,3], David H. Green[2], Aneika C. Leney[1]*

[1]School of Biosciences, University of Birmingham, Edgbaston, Birmingham, B15 2TT, UK.

[2]Scottish Association for Marine Science, Argyll, PA37 1QA, UK.

[3]Culture Collection of Algae and Protozoa (CCAP), Scottish Marine Institute, Oban, PA37 1QA, UK.

Table of Contents

Table S1: Molecular Weight Predictions of Phycobiliproteins from Different Species………S3

Methods; assignment of post-translational modifications……………………………………….S7

Methods; molecular weight assignments…………………………………………………………S7

Table S2: Molecular Weight Assignments of Phycobiliproteins………………………………..S8

Table S3: Allophycocyanin protein standard molecular weight assignments……………….S11

Figure S1: Native mass spectra show species dependent ‘fingerprints’…………………..…S12

Figure S2: Allophycocyanin level of detection by native mass spectrometry……………….S13

Figure S3: Cell counting of *Arthrospira maxima* (CCAP 1475/9) by light microscopy……..S14

References…………………………………………………………………………………………S14

Table S1: **Molecular Weight Predictions of Phycobiliproteins from Different Species**

Species	Phycobiliprotein	Sequence Length	Molecular weight (Da)*
**Allophycocyanin (APC) monomers**			
Anabaena cylindrica	APC alpha chain	160	17,081
Mastigocladus laminosus (Fischerella sp.)	APC alpha chain	160	17,121
Anabaena variabilis	APC beta chain	161	17,187
Synechocystis sp. (strain PCC 6803 / Kazusa)	APC beta chain	161	17,216
Synechococcus sp. (strain ATCC 27264 / PCC 7002 / PR-6) (Agmenellum quadruplicatum)	APC beta subunit	161	17,222
Synechocystis sp. (strain PCC 6714) (Aphanocapsa sp. (strain PCC 6714))	APC beta chain	161	17,243
Microchaete diplosiphon (Fremyella diplosiphon)	APC beta chain	162	17,269
Synechococcus sp. (strain ATCC 27264 / PCC 7002 / PR-6) (Agmenellum quadruplicatum)	APC alpha subunit	161	17,286
Nostoc sp. (strain PCC 7120 / SAG 25.82 / UTEX 2576)	APC subunit beta	162	17,305
Anabaena cylindrica	APC beta chain	161	17,316
Nostoc sp. (strain PCC 7120 / SAG 25.82 / UTEX 2576)	APC subunit alpha 1	161	17,346
Synechocystis sp. (strain PCC 6714) (Aphanocapsa sp. (strain PCC 6714))	APC alpha chain	161	17,355
Thermosynechococcus elongatus (strain BP-1)	APC beta chain	161	17,359
Microchaete diplosiphon (Fremyella diplosiphon)	APC alpha chain 1	161	17,365
Mastigocladus laminosus (Fischerella sp.)	APC beta chain	161	17,374
Arthrospira platensis (Spirulina platensis)	APC alpha chain	161	17,392
Synechococcus sp. (strain ATCC 27144 / PCC 6301 / SAUG 1402/1) (Anacystis nidulans)	APC beta chain	161	17,393
Synechocystis sp. (strain PCC 6803 / Kazusa)	APC alpha chain	161	17,412
Arthrospira platensis (Spirulina platensis)	APC beta chain	161	17,415
Synechococcus sp. (strain ATCC 27144 / PCC 6301 / SAUG 1402/1) (Anacystis nidulans)	APC alpha chain	161	17,422
Pyropia yezoensis (Susabi-nori) (Porphyra yezoensis)	APC beta chain	161	17,484
Pyropia yezoensis (Susabi-nori) (Porphyra yezoensis)	APC alpha chain	161	17,509
Thermosynechococcus elongatus (strain BP-1)	APC alpha chain	161	17,539
Synechococcus sp. (strain ATCC 27264 / PCC 7002 / PR-6) (Agmenellum quadruplicatum)	APC subunit alpha-B	161	17,702

Nostoc sp. (strain PCC 7120 / SAG 25.82 / UTEX 2576)	APC subunit alpha-B	161	17,811
Microchaete diplosiphon (Fremyella diplosiphon)	APC alpha chain 2	161	17,853
Synechocystis sp. (strain PCC 6803 / Kazusa)	APC subunit alpha-B	161	17,923
Synechococcus sp. (strain ATCC 27144 / PCC 6301 / SAUG 1402/1) (Anacystis nidulans)	APC alpha-B chain	163	18,130
Nostoc sp. (strain PCC 7120 / SAG 25.82 / UTEX 2576)	APC subunit beta-18	169	18,636
Synechococcus sp. (strain ATCC 27264 / PCC 7002 / PR-6) (Agmenellum quadruplicatum)	APC subunit beta-18	169	18,724
Synechocystis sp. (strain PCC 6803 / Kazusa)	APC subunit beta-18	169	18,892
**Allophycocyanin (APC) dimers**			
Anabaena cylindrica	APC dimer	-	34,397
Mastigocladus laminosus (Fischerella sp.)	APC dimer	-	34,495
Synechococcus sp. (strain ATCC 27264 / PCC 7002 / PR-6) (Agmenellum quadruplicatum)	APC dimer	-	34,508
Synechocystis sp. (strain PCC 6714) (Aphanocapsa sp. (strain PCC 6714))	APC dimer	-	34,598
Synechocystis sp. (strain PCC 6803 / Kazusa)	APC dimer	-	34,628
Arthrospira platensis (Spirulina platensis)	APC dimer	-	34,807
Synechococcus sp. (strain ATCC 27144 / PCC 6301 / SAUG 1402/1) (Anacystis nidulans)	APC dimer	-	34,815
Thermosynechococcus elongatus (strain BP-1)	APC dimer	-	34,898
Pyropia yezoensis (Susabi-nori) (Porphyra yezoensis)	APC dimer	-	34,993
Nostoc sp. (strain PCC 7120 / SAG 25.82 / UTEX 2576)	APC dimer	-	35,116
Microchaete diplosiphon (Fremyella diplosiphon)	APC dimer	-	35,122
**Phycocyanin (PC) monomers**			
Microchaete diplosiphon (Fremyella diplosiphon)	C-PC-1 alpha subunit	162	17,228
Pseudanabaena tenuis (strain PCC 7409)	C-PC-1 alpha subunit	162	17,233
Synechococcus sp. (strain ATCC 27144 / PCC 6301 / SAUG 1402/1) (Anacystis nidulans)	C-PC-2 alpha subunit	163	17,258
Synechococcus sp. (strain ATCC 27144 / PCC 6301 / SAUG 1402/1) (Anacystis nidulans)	C-PC-1 alpha subunit	163	17,288 #
Synechococcus elongatus (strain PCC 7942 / FACHB-805) (Anacystis nidulans R2)	C-PC alpha subunit	163	17,288 #
Pseudanabaena tenuis (strain PCC 7409)	C-PC-2 alpha subunit	162	17,301
Synechococcus sp. (strain WH7803)	R-PC alpha subunit	162	17,320
Synechococcus sp. (strain WH8103)	R-PC-2 subunit alpha	162	17,335

Synechococcus sp. (strain WH8020)	R-PC-2 subunit alpha	162	17,369
Microchaete diplosiphon (Fremyella diplosiphon)	C-PC-3 alpha subunit	162	17,393
Thermosynechococcus elongatus (strain BP-1)	C- PC alpha subunit	162	17,443
Synechocystis sp. (strain PCC 6701)	C- PC alpha subunit	163	17,452
Nostoc sp. (strain PCC 7120 / SAG 25.82 / UTEX 2576)	C-PC alpha subunit	163	17,458
Microchaete diplosiphon (Fremyella diplosiphon)	C-PC-2 alpha subunit	162	17,466
Mastigocladus laminosus (Fischerella sp.)	C-PC alpha subunit	163	17,524
Synechocystis sp. (strain PCC 6803 / Kazusa)	C-PC alpha subunit	162	17,587
Arthrospira platensis (Spirulina platensis)	C-PC alpha subunit	162	17,602
Synechococcus sp. (strain ATCC 27264 / PCC 7002 / PR-6) (Agmenellum quadruplicatum)	C-PC subunit alpha	162	17,622
Pseudanabaena tenuis (strain PCC 7409)	C-PC-1 beta subunit	172	17,881
Synechococcus sp. (strain WH7803)	R-PC beta subunit	172	17,909
Microchaete diplosiphon (Fremyella diplosiphon)	C-PC-1 beta subunit	172	17,920
Synechococcus sp. (strain WH8103)	R-PC-2 beta chain	172	17,943
Synechococcus sp. (strain WH8020)	R-PC-2 beta chain	172	18,056
Arthrospira platensis (Spirulina platensis)	C-PC beta subunit	172	18,094
Pseudanabaena tenuis (strain PCC 7409)	C-PC-2 beta subunit	172	18,114
Microchaete diplosiphon (Fremyella diplosiphon)	C-PC-2 beta subunit	172	18,122
Synechocystis sp. (strain PCC 6803 / Kazusa)	C-PC beta subunit	172	18,126
Thermosynechococcus elongatus (strain BP-1)	C-PC beta subunit	172	18,187
Microchaete diplosiphon (Fremyella diplosiphon)	C-PC-3 beta subunit	173	18,237
Synechococcus sp. (strain ATCC 27144 / PCC 6301 / SAUG 1402/1) (Anacystis nidulans)	C-PC-1 beta subunit	173	18,270 #
Synechococcus elongatus (strain PCC 7942 / FACHB-805) (Anacystis nidulans R2)	C-PC beta subunit	173	18,270 #
Synechococcus sp. (strain ATCC 27144 / PCC 6301 / SAUG 1402/1) (Anacystis nidulans)	C-PC-2 beta subunit	173	18,298
Synechocystis sp. (strain PCC 6701)	C-PC beta subunit	172	18,323
Synechococcus sp. (strain ATCC 27264 / PCC 7002 / PR-6) (Agmenellum quadruplicatum)	C-PC subunit beta	172	18,336
Nostoc sp. (strain PCC 7120 / SAG 25.82 / UTEX 2576)	C-PC beta subunit	173	18,387
Mastigocladus laminosus (Fischerella sp.)	C-PC beta subunit	173	18,521

**Phycocyanin (PC) dimers**			
Pseudanabaena tenuis (strain PCC 7409)	C-PC-1 dimer	-	35,114
Microchaete diplosiphon (Fremyella diplosiphon)	C-PC-1 dimer	-	35,148
Synechococcus sp. (strain WH7803)	R-PC dimer	-	35,229
Synechococcus sp. (strain WH8103)	R-PC dimer	-	35,278
Pseudanabaena tenuis (strain PCC 7409)	C-PC-2 dimer	-	35,415
Synechococcus sp. (strain WH8020)	R-PC dimer	-	35,425
Synechococcus sp. (strain ATCC 27144 / PCC 6301 / SAUG 1402/1) (Anacystis nidulans)	C-PC-2 dimer	-	35,556
Synechococcus sp. (strain ATCC 27144 / PCC 6301 / SAUG 1402/1) (Anacystis nidulans)	C-PC-1 dimer	-	35,558 #
Synechococcus elongatus (strain PCC 7942 / FACHB-805) (Anacystis nidulans R2)	C-PC dimer	-	35,558 #
Microchaete diplosiphon (Fremyella diplosiphon)	C-PC-2 dimer	-	35,588
Microchaete diplosiphon (Fremyella diplosiphon)	C-PC-3 dimer	-	35,630 #
Thermosynechococcus elongatus (strain BP-1)	C-PC dimer	-	35,630 #
Arthrospira platensis (Spirulina platensis)	C-PC dimer	-	35,696
Synechocystis sp. (strain PCC 6803 / Kazusa)	C-PC dimer	-	35,713
Synechocystis sp. (strain PCC 6701)	C-PC dimer	-	35,775
Nostoc sp. (strain PCC 7120 / SAG 25.82 / UTEX 2576)	C-PC dimer	-	35,845
Synechococcus sp. (strain ATCC 27264 / PCC 7002 / PR-6) (Agmenellum quadruplicatum)	C-PC dimer	-	35,958
Mastigocladus laminosus (Fischerella sp.)	C-PC dimer	-	36,045

* Molecular weights have been calculated from the sequence excluding any post-translational modifications.
# indicates molecular weights that match that of another species. In these cases, alternate phycocyanin monomer chains are present that would form unique phycocyanin dimers, thus ensuring these species could be distinguished from one another by native mass spectrometry.

Post-translational Modifications

Based on data from Uniprot[1], the α and β subunits of allophycocyanin and phycocyanin are post-translationally modified to various extents with phycocyanobilin (PCB), N4-methylasparagine and N-terminal methionine removal. These modifications were, therefore, accounted for when calculating the theoretical molecular weights of the phycobiliprotein complexes. For the phycocyanin α subunit, we assumed the addition of one PCB (+585.7 Da), whilst for the corresponding β subunit, we assumed the addition of two PCBs and the modification of Asp72 to N4-methylAsp72 (+14.0 Da). For the allophycocyanin α subunit, we included the addition of one PCB, whilst for the allophycocyanin β subunit, we included the addition of one PCB and the modification of Asp71 to N4-methylAsp71. For both phycocyanin and allophycocyanin, we assumed the removal of the initiator Methionine (-131.2 Da) was between 0 and 1 for each subunit.

Molecular Weight Assignments

Table S1 show the experimentally determined molecular weights of phycocyanin and allophycocyanin for all species variants analysed. The amino acid sequences as determined from metagenomics data were used to calculate the theoretical molecular weight of the allophycocyanin and phycocyanin complexes, assuming the presence of predicted post-translational modifications (PTMs) mentioned above. 'ND' indicates protein complexes that were not detected. Similarly, Table S2 shows the experimentally determined molecular weights of allophycocyanin for the allophycocyanin purified protein standard whereby the metagenomics data for *Arthrospira maxima* were used to calculate the theoretical molecular weights of the complexes.

Table S2: Molecular Weight Assignments of Phycobiliproteins

Species	Theoretical molecular weight (Da)	PCB	N4-methylAsp	N-terminal methionine removal	Experimental molecular weight (Da)	Mass deviation
**Phycocyanin dimer (αβ)**						
Spirulina major (CCAP 1475/3)	37,304	3	1	-	37,303.7 +/- 0.4	0.0008%
Coccochloris elabens (CCAP 1413/1) **Synonyms:** *Aphanothece elabens*	38,014.8	3	1	-	38,015.3 +/- 0.0	0.001%
Spirulina subsalsa (CCAP 1475/1)	36,775.6	3	1	-	36,776.6 +/- 0.2	0.003%
Gloeocapsopsis crepidinum (CCAP 1425/1)	37,874.7	3	1	-	37,874.1 +/- 0.3	0.002%
Nodularia harveyana (CCAP 1452/1)	37,530.2	3	1	2	37,529.9 +/- 0.0	0.0008%
Chroococcus sp. (CCAP 1412/6)	37,628.6	3	1	-	37,631.2 +/- 0.3	0.007%
Oscillatoria nigroviridis (CCAP 1459/9)	37,689.5	3	1	-	37,691.2 +/- 0.3	0.005%
Arthrospira maxima (CCAP 1475/9) **Synonyms:** *Limnospira maxima*	37,464.5	3	1	-	37,467.1 +/- 0.5	0.007%
Euhalothece sp. (CCAP 1421/1)	37,484.1	3	1	-	37,484.2 +/- 0.7	0.0003%
**Phycocyanin hexamer ($\alpha_3\beta_3$)**						
Spirulina major (CCAP 1475/3)	111,912	9	3	-	111,932 +/- 10	0.02%

Coccochloris elabens (CCAP 1413/1) **Synonyms:** *Aphanothece elabens*	114,044	9	3	-	114,111 +/- 9	0.06%
Spirulina subsalsa (CCAP 1475/1)	110,327	9	3	-	110,341 +/- 7	0.01%
Gloeocapsopsis crepidinum (CCAP 1425/1)	113,624	9	3	-	113,663 +/- 6	0.03%
Nodularia harveyana (CCAP 1452/1)	112,591	9	3	6	ND	-
Chroococcus sp. (CCAP (1412/6)	112,886	9	3	-	112,933 +/- 4	0.04%
Oscillatoria nigroviridis (CCAP 1459/9)	113,069	9	3	-	113,125 +/- 2	0.05%
Arthrospira maxima (CCAP 1475/9) **Synonyms:** *Limnospira maxima*	112,394	9	3	-	112,435 +/- 6	0.04%
Euhalothece sp. (1421/1)	112,452	9	3	-	112,523 +/- 10	0.06%
**Allophycocyanin dimer (αβ)**						
Spirulina major (CCAP 1475/3)	35,455.4	2	1	1	ND	-
Coccochloris elabens (CCAP 1413/1) **Synonyms:** *Aphanothece elabens*	35,999	2	1	1	ND	-
Spirulina subsalsa (CCAP 1475/1)	35,547.5	2	1	1	35,546.6 +/- 0.4	0.003%
Gloeocapsopsis crepidinum (CCAP 1425/1)	36,147.1	2	1	1	ND	-

Nodularia harveyana (CCAP 1452/1)	35,716.6	2	1	1	35,715.8 +/- 0.5	0.002%
Chroococcus sp. (CCAP 1412/6)	36,084	2	1	1	ND	-
Oscillatoria nigroviridis (CCAP 1459/9)	35,941	2	1	1	ND	-
Arthrospira maxima (CCAP 1475/9) **Synonyms:** *Limnospira maxima*	35,775.8	2	1	1	ND	-
Euhalothece sp. (CCAP 1421/1)	35,681.7	2	1	1	ND	-
**Allophycocyanin hexamer ($\alpha_3\beta_3$)**						
Spirulina major (CCAP 1475/3)	106,366	6	3	3	106,385 +/- 3	0.02%
Coccochloris elabens (CCAP 1413/1) **Synonyms:** *Aphanothece elabens*	107,997	6	3	3	108,068 +/- 10	0.07%
Spirulina subsalsa (CCAP 1475/1)	106,643	6	3	3	106,652 +/- 2	0.008%
Gloeocapsopsis crepidinum (CCAP 1425/1)	108,441	6	3	3	108,477 +/- 3	0.03%
Nodularia harveyana (CCAP 1452/1)	107,150	6	3	3	107,171 +/- 1	0.02%
Chroococcus sp. (CCAP 1412/6)	108,252	6	3	3	ND	-
Oscillatoria nigroviridis (CCAP 1459/9)	107,823	6	3	3	107,865 +/- 3	0.04%
Arthrospira maxima (CCAP 1475/9)	107,327	6	3	3	107,348 +/- 2	0.02%

**Synonyms:** *Limnospira maxima*						
Euhalothece sp. (CCAP 1421/1)	107,045	6	3	3	ND	-

Table S3: Allophycocyanin protein standard molecular weight assignments

Protein species	Theoretical molecular weight (Da)	PCB	N4-methylAsp	Methionine removal	Experimental molecular weight (Da)	Mass deviation
α monomer	17,846.3	1	-	1	17,846.2 +/- 0.0	0.0007%
β monomer	17,929.5	1	1	-	17,929.1 +/- 0.1	0.002%
Dimer (αβ)	35,775.8	2	1	1	35,774.5 +/- 0.4	0.004%
Hexamer ($\alpha_3\beta_3$)	107,327.4	6	3	3	107,377 +/- 3	0.05%

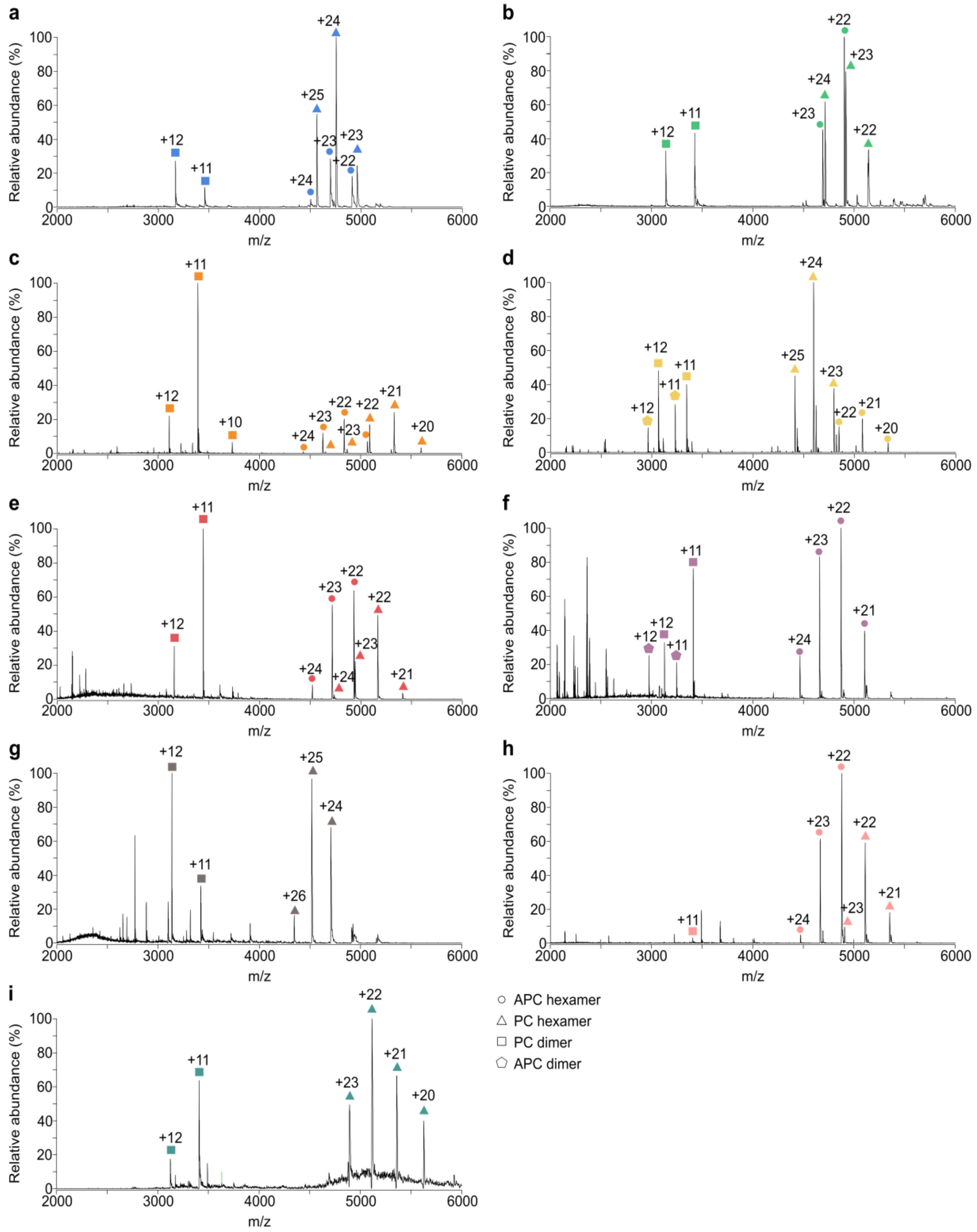

Figure S1: Native mass spectra show species dependent 'fingerprints'. Native MS of lysates from *Coccochloris elabens* (CCAP 1413/1) (a), *Oscillatoria nigroviridis* (CCAP 1459/9) (b), *Spirulina major* (CCAP 1475/3) (c), *Spirulina subsalsa* (CCAP 1475/1) (d), *Gloeocapsopsis crepidinum* (CCAP 1425/1) (e), *Nodularia harveyana* (CCAP 1452/1) (f), *Chroococcus sp.* (CCAP 1412) (g), *Arthrospira maxima* (CCAP 1475/9) (h) and *Euhalothece sp.* (CCAP 1421/1) (i); showing allophycocyanin hexamer (circle), allophycocyanin dimer (pentagon), phycocyanin hexamer (triangle) and phycocyanin dimer (square) as the pre-dominant protein complexes detected. The phycobiliprotein peaks present are coloured and labelled.

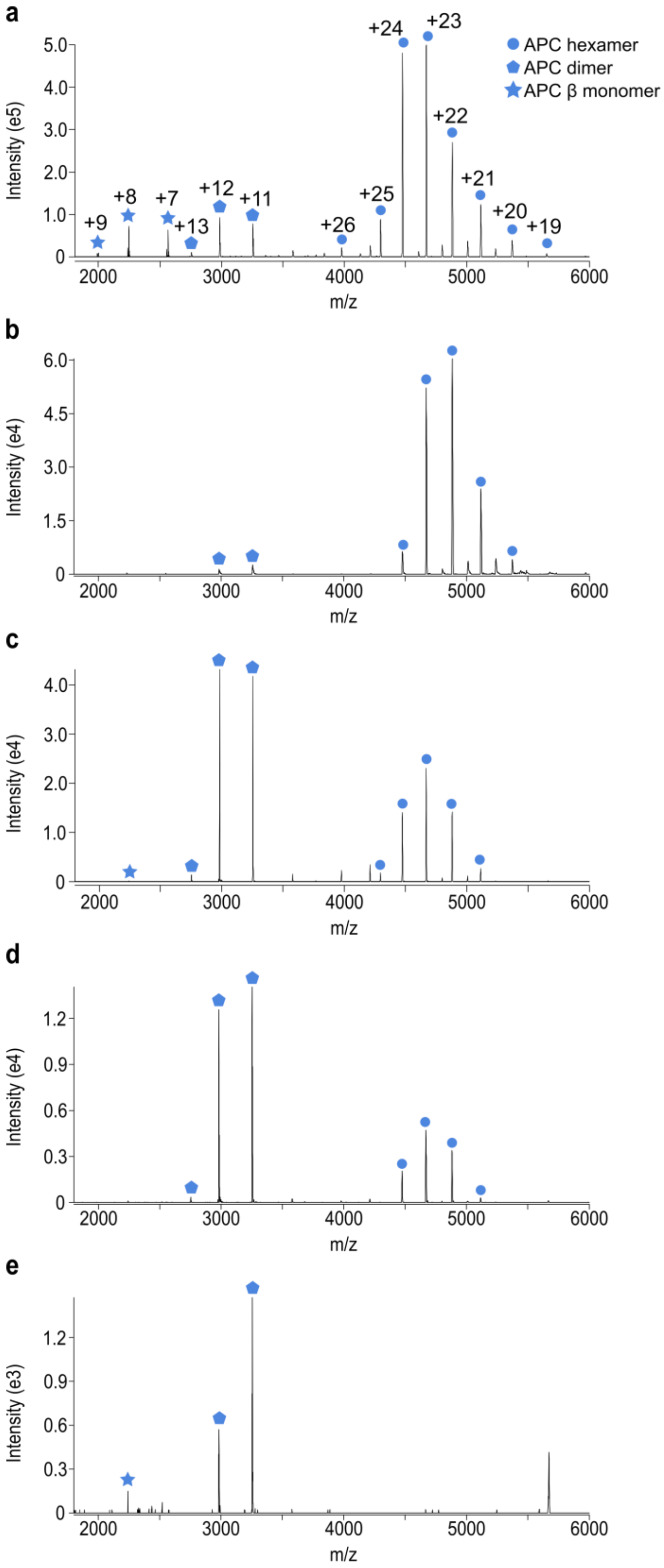

Figure S2: Allophycocyanin level of detection by native mass spectrometry. Native MS of purified allophycocyanin at 50 mg/L (a), 10 mg/L (b), 5 mg/L (c), 2.5 mg/L (d) and 1.26 mg/L (e). The allophycocyanin hexamer (circle), dimer (pentagon) and the monomeric β subunit (star) are shown.

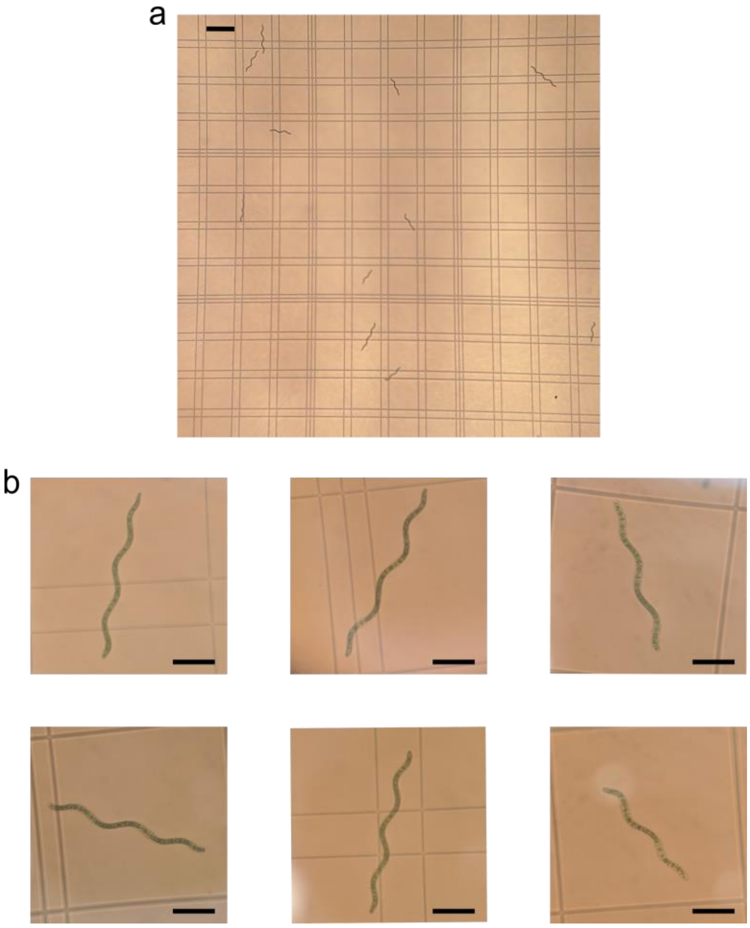

Figure S3: Cell counting of *Arthrospira maxima* (CCAP 1475/9) by light microscopy. Typical observation at 500,000 cells/mL whereby the number of filaments per mL were determined (a) and this multiplied by the number of cells per filament to obtain the overall cell count. Example images of the algae filaments are shown in (b). The scale bars represent 0.2 mm and 0.05 mm for a) and b), respectively.

REFERENCES

1. The UniProt Consortium. UniProt: the universal protein knowledgebase in 2021. *Nucleic Acids Res.* **2021**, 49, D480–D489.
